# Molecular Detection of Kinetoplastid Species in Ticks and Fleas Associated with Dogs and Humans in Mexico

**DOI:** 10.3390/pathogens13121072

**Published:** 2024-12-06

**Authors:** Héctor M. Zazueta-Islas, Beatriz Salceda-Sánchez, Herón Huerta-Jiménez, Carlos I. Miranda-Caballero, Marlene Solis-Cortés, Yaretzi de la Cruz-Pacheco, Ana Cristina Luquín-García, Laura V. Mondragon-Peña, Jair Reyes-Hernández, José L. Bravo-Ramos, María-Guadalupe Sánchez-Otero, Javier C. Huerta-Peña, Rosa I. Hernández-Herrera, Pablo San Martin-del Angel, André Luiz Rodrigues Roque, Ángel Rodríguez-Moreno, Víctor Sánchez-Cordero, Héctor Abelardo Rodríguez Martínez, Estefania Grostieta, Ingeborg Becker, Sokani Sánchez-Montes

**Affiliations:** 1 Centro de Medicina Tropical, División de Investigación, Facultad de Medicina, Universidad Nacional Autónoma de México, Mexico City 04510, Mexico; manuzazueta@ciencias.unam.mx (H.M.Z.-I.); miranda-caballero@ciencias.unam.mx (C.I.M.-C.); marlenesolis@ciencias.unam.mx (M.S.-C.); 2 Laboratorio de Entomología, Instituto de Diagnóstico y Referencia Epidemiológicos, Secretaría de Salud, Mexico City 01480, Mexico; betysal_2000@yahoo.com (B.S.-S.); cerato_2000@yahoo.com (H.H.-J.); 3 Facultad de Ciencias Biológicas y Agropecuarias, Región Poza Rica-Tuxpan, Universidad Veracruzana, Tuxpan de Rodríguez Cano, Veracruz 92870, Mexico; zs19008315@estudiantes.uv.mx (Y.d.l.C.-P.); zs24018925@estudiantes.uv.mx (A.C.L.-G.); zs19008304@estudiantes.uv.mx (L.V.M.-P.); zs19008267@estudiantes.uv.mx (J.R.-H.); jhuerta@uv.mx (J.C.H.-P.); 4 Facultad de Bioanálisis, Región Veracruz, Universidad Veracruzana, Veracruz 91700, Mexico; jbravo@uv.mx (J.L.B.-R.); guadsanchez@uv.mx (M.-G.S.-O.); 5 Laboratorio de Biotecnología Ambiental, Facultad de Ciencias Biológicas y Agropecuarias, Región Poza Rica-Tuxpan, Universidad Veracruzana, Tuxpan de Rodríguez Cano, Veracruz 92870, Mexico; idhernandez@uv.mx (R.I.H.-H.); pmartin@uv.mx (P.S.M.-d.A.); 6 Laboratório de Biologia de Tripanosomatídeos, Instituto Oswaldo Cruz/FIOCRUZ, Rio de Janeiro 21040-900, Brazil; roque@ioc.fiocruz.br; 7 Laboratorio de Geografía de la Biodiversidad, Pabellón Nacional de la Biodiversidad Instituto de Biología, Universidad Nacional Autónoma de México, Mexico City 04510, Mexico; tanicandil@hotmail.com (Á.R.-M.); victor@ib.unam.mx (V.S.-C.); 8 Laboratorio de Investigaciones Anatomopatológicas, Unidad de Medicina Experimental, Facultad de Medicina, Universidad Nacional Autónoma de México, Mexico City 04510, Mexico; harodriguezm@yahoo.com.mx

**Keywords:** ectoparasites, *Rhipicephalus sanguineus* s.l., *Blechomonas lauriereadi*, *Parabodo* sp., *Trypanosoma caninum*

## Abstract

The Trypanosomatidae family encompasses around 24 genera of unicellular protozoans, many of which are transmitted by various hematophagous arthropods, particularly members of the Orders Diptera and Hemiptera. Fleas and ticks—an understudied group of ectoparasites—have been shown to be hosts of a wide and crescent variety of trypanosomatid species. Further, fleas and ticks of companion animals have been particularly neglected in trypanosomatid surveillance despite the proximity to human populations and the anthropophagous habits of many of these arthropods, which can potentially act as vectors of zoonotic trypanosomatids. We aimed to identify the presence, characterize the species, and establish the prevalence of Kinetoplastids, including members of the Trypanosomatidae family, in ectoparasites collected from dogs and humans from Mexico. A total of 537 ectoparasite specimens belonging to six ectoparasite taxa (*Amblyomma mixtum*, *A. tenellum*, *Ctenocephalides felis felis*, *Pulex simulans*, *Rhipicephalus linnaei*, and *Rh. sanguineus* s.s.) were collected from 15 States of Mexico. An 800 bp fragment of the 18S-rDNA gene from kinetoplastids was amplified and sequenced. The presence of two agents (*Trypanosoma caninum* and *Parabodo* sp.) was detected in *R. linnaei* ticks and one (*Blechomonas lauriereadi*) in the cat flea *Ct. felis felis*. This is the first record of genetic material of kinetoplastid species in ectoparasites from dogs and humans in Mexico.

## 1. Introduction

The Trypanosomatidae family encompasses around 24 genera of unicellular protozoans that show an elongated morphology, the presence of a unique flagellum, and a parasitic life cycle, which can be monoxenous in invertebrate hosts, or alternate between invertebrate and vertebrate hosts or plants [1,2,3]. Several genera contain species of importance for public health and veterinary medicine, such as members of the genera *Leishmania* and *Trypanosoma*, which are the etiological agents of the zoonotic tropical diseases leishmaniasis and Chagas disease, respectively [4].

The heteroxenous representatives of these trypanosomatids are transmitted by various groups of arthropods, such as the Orders Diptera and Hemiptera [1,5]. Some efforts have recently been made to identify the diversity of trypanosomatids associated with other groups of ectoparasites, such as members of the orders Siphonaptera and Ixodida (fleas and ticks, respectively) [6,7,8].

In the last 20 years, researchers have identified a diverse and complex array of trypanosomatids associated with fleas, primarily belonging to the genera *Trypanosoma* (subgenus *Herpetosoma*), *Leptomonas*, and *Blechomonas* [6,8,9,10]. These trypanosomatids are generally considered to be specialized flea parasites, with *Trypanosoma* (*Herpetosoma*) species known to be transmitted to vertebrate hosts, such as rodents and lagomorphs. Some species have regained significance from a public health perspective, including *Trypanosoma lewisi*, an agent of an opportunistic rat- and flea-borne human emergent zoonosis in southeast Asia [11]. Further, a recent review showed a wide range of records of trypanosomatids associated with ticks, mainly with members of the family Ixodidae, commonly known as hard ticks [12]. The presence of six trypanosomatid species associated with 20 ticks in three zoogeographic regions were identified, highlighting the presence of parasites responsible for human cutaneous and visceral leishmaniasis, as well as African trypanosomiasis. In the American continent, studies on the identification of trypanosomatid species associated with ticks have been conducted only in Argentina, Brazil, Chile, Mexico and Peru, revealing seven trypanosomatid species linked to six hard ticks of the genera *Amblyoma* and *Rhipicephalus* [13,14,15,16,17,18,19,20,21,22,23,24,25,26,27,28].

However, trypanosomatids in ectoparasites associated with dogs, cats and humans have remained virtually unexplored. Since ticks and fleas are the two primary groups of ectoparasites infesting dogs and cats—companion animals of humans—it is important to monitor the trypanosomatid species associated with these ectoparasites. Such monitoring is essential not only to prevent the transmission of potentially zoonotic trypanosomatid species, but also to establish a comprehensive biological inventory of the species circulating among ectoparasites of these companion animals. Further, DNA of two species of the *Trypanosoma* genus (*Trypanosoma cruzi* and *Trypanosoma evansi*) and two of the *Leishmania* genus (*Leishmania brasiliensis* and *Leishmania infantum*) were detected in four species of hard ticks (*Amblyomma cajennense*, *Amblyomma tigrinum* and *Rhipicephalus sanguineus* s.s.) parasitizing dogs in Brazil and Chile [16,17,18,19,20,21,27]. Other studies have recorded *T. lewisi* and *T. cruzi* DNA in rodent flea guts from Venezuela [29], and a negative report of the presence of *T. cruzi* in three dog flea species (*Ctenocephalides canis*, *Ctenocephalides felis felis*, and *Pulex irritans*) in Chile [27]. Here, we aimed to document the presence and determine the prevalence of kinetoplastids, including members of the Trypanosomatidae family, in ectoparasites that infest dogs and humans from a wide geographical range in Mexico.

## 2. Materials and Methods

**Figure 1:**
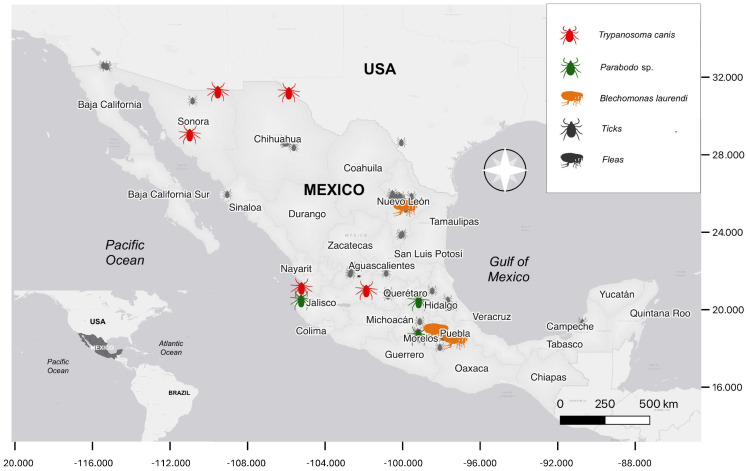
Sampling sites of ectoparasites of dogs and humans selected for kinetoplastid molecular detection in Mexico.

As part of the routine detection of *Rickettsia* by the National Network of Public Health Laboratories of the Ministry of Health and private veterinary clinics, ectoparasites from 76 localities in 49 municipalities, including 15 States of Mexico, were collected (Table 1, Figure 1).

215 dogs and 1 cat were externally examined for ectoparasites; additionally, ticks were manually collected from 8 persons who were incidentally bitten (Table 1). Ectoparasites were then placed in 1.5 mL polypropylene conical tubes containing 70% ethanol. The samples were transported to the Laboratorio de Entomologia from Instituto de Diagnóstico y Referencia Epidemiológicos and examined for morphological identification using a Carl Zeiss Stemi 305 stereomicroscope and an Axiocam 208 camera. Identification was performed using specialized taxonomic keys from SENASICA [30] and Bermudez et al. [31] for ticks and Salceda-Sánchez [32] for fleas.

Genomic DNA was extracted from individual specimens, which were macerated using a new pestle for each sample to prevent cross-contamination and the commercial kit DNeasy Blood & Tissue Kit, (QIAGEN, Inc., Hilden, Germany), using the protocol for purification of total DNA from animal tissues, with an elution in a final volume of 100 µL. Once the DNA was recovered, it was frozen at −20 °C until use.

As an endogenous control of extraction and for the molecular identification of ticks of the *Rhipicephalus sanguineus* complex [33], a 400 bp segment of the *16S-rDNA* ribosomal gene was amplified using the primers 16S − 1 (5′-CTGCTCAATGATTTTTTAAATTGCTGTGG-3′) and 16S + 2 (5′-CGCTGTTATCCCTAGAGTATT-3′) and thermal conditions described by Norris et al. [34]. The same procedure was repeated for the cytochrome oxidase subunit I (*COI*) of fleas using the primers LCO1-1490 (5′-GGTCAACAAATCATAAAGATATTG -3′) and HCO1-2198 (5′-TAAACTTCAGGGTGACCAAAAAATCA-3′) following the thermal conditions described by Folmer et al. [35].

For the detection of kinetoplastids, including those species of the Trypanosomatidae family, a fragment of 800 bp of the 18S small subunit of the ribosomal gene (*SSU rDNA*) was amplified using oligonucleotides 609F (5′-CACCCGCGGTAATTCCAGC-3′) and 706R (5′-CTGAGACTGTAACCTCAA-3′) [36], following the thermal conditions described by Borghesan et al. [37]. DNA from the ectoparasite samples was analyzed by conventional PCR by pooling three to five specimens of the same species from the same collection period and locality in a total of 80 pools. The reaction mixture consisted of 12.5 μL of GoTaq^®^ Green Master Mix (2X) from Promega Corporation (Madison, WI, USA), 1 µL of each oligonucleotide (2 µM each), 6.5 µL of DNase-free water, and 4 µL of DNA (The total DNA was quantified using a NanoDrop 2000/2000c spectrophotometer (Thermo Fisher Scientific, Waltham, MA, USA) and subsequently adjusted to a concentration of 200–300 ng) in a final volume of 25 μL. Positive (DNA from *Rhiphicephalus microplus*, *Ctenocephalies felis felis*, and *T. cruzi*) and negative controls (nuclease-free water) were included. Amplicons were visualized on 2% agarose gels stained with Syto 60 fluorochrome using TAE buffer at 85 V for 45 min.

The positive PCR products were sent to Macrogen^®^ (Seoul, Republic of Korea), for sequencing using the BigDye Terminator v3.1 Cycle Sequencing Kit (Cat. 4337455, Thermo Fisher, Waltham, MA, USA). The samples were purified by the BigDye XTerminator (Cat. 4376486, Thermo Fisher, Waltham, MA, USA) prior to loading on the ABI 3730 × L DNA analyzer (Thermo Fisher, Waltham, MA, USA).

The resulting sequences were compared with references deposited in GenBank using the BLASTn tool. Subsequently, a global alignment was performed using the Clustal W algorithm, and phylogenetic reconstruction was carried out based on the Maximum Likelihood inference method in IQ-TREE [38] with 10,000 bootstrap replicates, eliminating any gaps during the analysis.

## 3. Results

**Figure 2:**
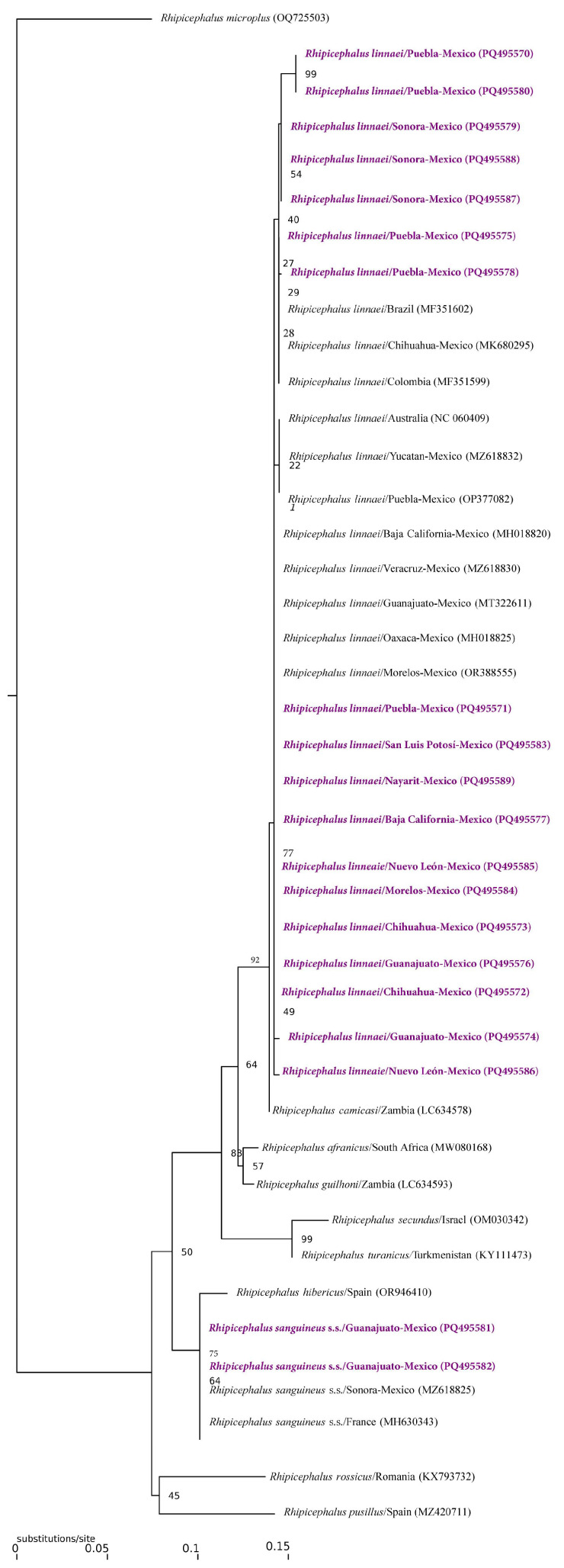
Maximum Likelihood inference phylogenetic compress analysis of the selected 16S rDNA partial gene for *Rhipicephalus sanguineus* s.s. and *Rhipicephalus linnaei* collected in the present study.

A total of 537 ectoparasites were collected. These included four species of hard ticks: 422 (214♀, 198♂ and 10 nymphs) specimens of *Rhipicephalus linnaei*, 19 (5♀, 9♂, 5 nymphs) speciments of *Rhipicephalus sanguineus* s.s., 14 (5♀, 5♂, 4 nymphs) specimens of *Amblyomma mixtum* and 11 (10♀, 1♂) specimens of *Amblyomma tenellum*. Furthermore, 66 (42♀, 24♂) fleas of *Ctenocephalides felis felis* species and 5 (4♀, 1♂) fleas of *Pulex simulans* were collected from 215 hosts, including 206 dogs, 1 cat, and 8 persons from 76 localities of 49 municipalities of 15 States of Mexico (including Aguascalientes, Baja California, Campeche, Chihuahua, Guanajuato, Hidalgo, Jalisco, Mexico City, Morelos, Nayarit, Nuevo León, Puebla, San Luis Potosí, Sinaloa, Sonora) (Table 1). The recovered sequences confirmed the morphological identification of three tick species, exhibiting a similarity of 99.51% (404/406 bp), 99.05% (420/424 bp) and 97.80% (444/454 bp) with sequences of *Rh. linnaei*, *Rh. sanguineus* s.s. and *A. mixtum* from Cuba, Germany, and USA (GenBank accession numbers KP830114.1, OP326204.1 and KM519935.1, respectively). The phylogenetic reconstruction supported the identification of the members of the *R. sanguineus* complex, grouping our sequences with those of references of *R. linnaei*, and *R. sanguineus* s.s. deposited in GenBank in two monophyletic groups, with support values above 90 (Figure 2).

**Figure 3:**
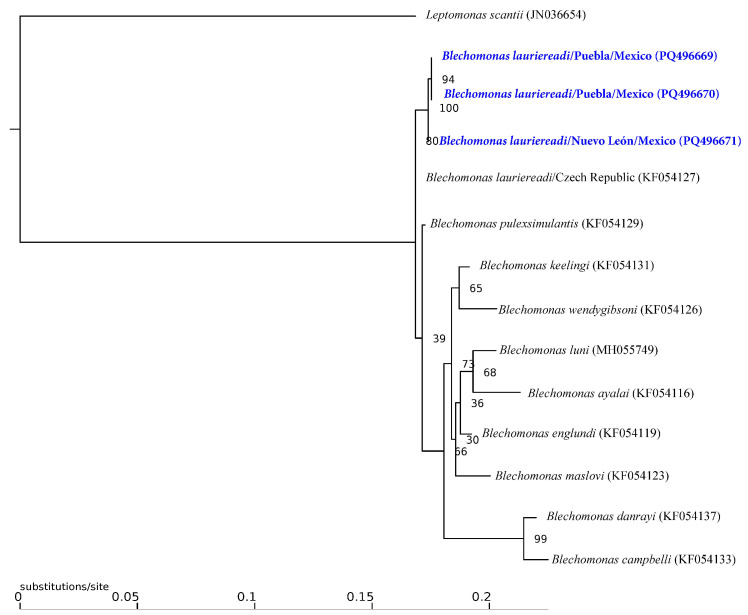
Maximum Likelihood inference phylogenetic compress analysis of the selected 18S rDNA partial gene for several members of the genus *Blechomonas*. The sequences obtained in the present study are presented in bold and blue.

**Figure 4:**
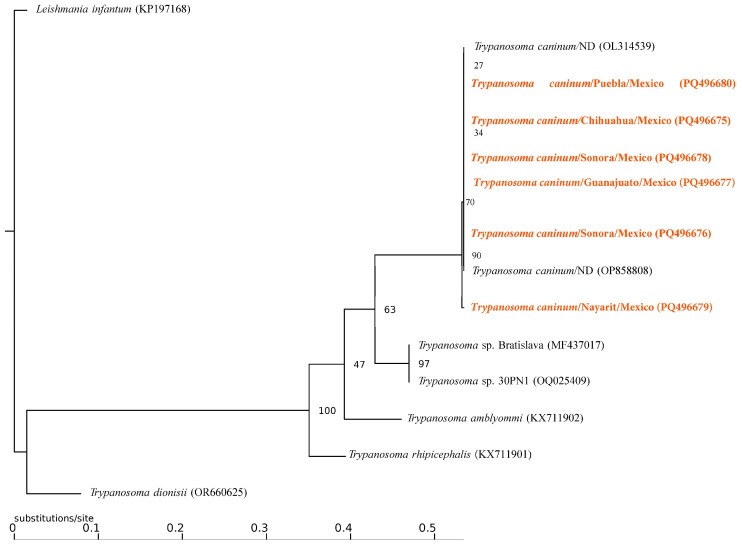
Maximum Likelihood inference phylogenetic compress analysis of the selected 18S rDNA partial gene for several members of the genus *Trypanosoma*. The sequences obtained in the present study are presented in bold and orange.

Of the 537 ectoparasites grouped into 80 pools, only 15% (12 pools: 3 of *Ct. felis felis* and 9 of *Rh. linnaei*) recovered from Chihuahua, Guanajuato, Nayarit, Nuevo León, Puebla, and Sonora tested positive for the presence of Kinetoplastid DNA (Table 1). The molecular identification of the recovered sequences confirmed the presence of three taxa: (1) *Trypanosoma caninum* in six pools of *R. linnaei* from Nuevo León, Puebla and Sonora with a similarity of 99% with sequences from Brazil (KF805453.1); (2) *Parabodo* sp. in three pools of *R. linnaei* from Jalisco and Morelos with a similarity of 96% from sequences of *Parabodo caudatus* from Germany (JF754435.1), and (3) *Blechomonas lauriereadi* in three pools from *Ct. felis felis* from Nuevo León and Puebla with a similarity of 99% from a sequence from Czech Republic (KF054127.1). The Maximum Likelihood analysis confirms that the trypanosomatid species corresponded to *Trypanosoma caninum* and *Blechomonas lauriereadi*, showing support values above 90 (Figure 3 and Figure 4).

**Figure 5:**
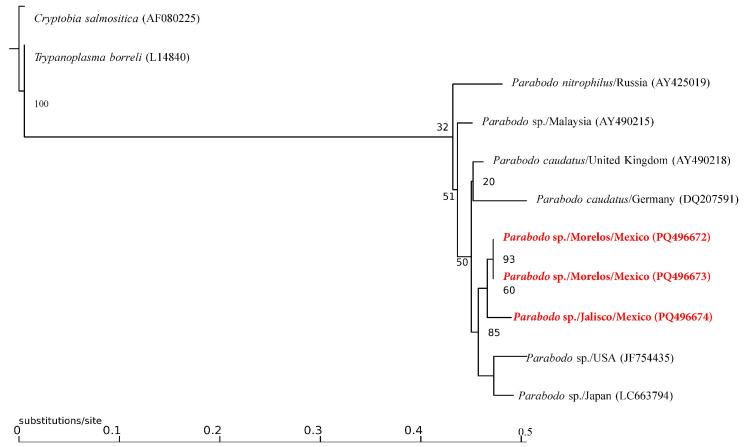
Maximum Likelihood inference phylogenetic compress analysis of the selected 18S rDNA partial gene for several members of the genus *Parabodo*. The sequences obtained in the present study are presented in bold and red.

Due to the low similarity of the recovered *Parabodo* sequences, as well as the branch length of the phylogenetic reconstruction (Figure 5), it is possible to assume that this may represent a still not described species within the genus.

Sequences were deposited in GenBank under the following accession numbers: Trypanosomatids (PQ496669- PQ496680) and *Rhipicephalus sanguineus* s.l. (PQ495570-PQ495590).

## 4. Discussion

The study of trypanosomatids associated with ticks has gained interest in the last decades [7,14,15,16,17,18,19,20,21,22,23,24,25,26,27,28]. At least six species of trypanosomatids associated with 20 species of ticks have been identified worldwide, where most of the studies are concentrated in the Afrotropical and Palearctic regions [12]. The monitoring of trypanosomatids in ticks associated with dogs is still limited. In the Neotropical region, only *T. cruzi* and *T. evansi* genetic material was detected in *Amblyomma cajennense*, *Amblyomma tigrinum* and *Rhipicephalus sanguineus* s.s. parasitizing dogs in Brazil and Chile [17,18,19,20,21,27]. The first study to identify *Trypanosoma* species in ticks from Mexico was conducted in haemolymph samples from *Rhipicephalus microplus* ticks associated with cattle from the State of Yucatan, in which the presence of *Trypanosoma theileri* was detected by light microscopy [14].

To our knowledge, our present work represents the first systematic approach to studying trypanosomatids from ticks associated with dogs and people in Mexico.

Although species of the *Rhipicephalus sanguineus* s.l. complex represent the group of ticks most widely monitored for the detection of pathogens in Mexico [39,40,41,42,43], it is also relevant to highlight that surveillance has focused primarily on the identification of species of the genera *Rickettsia*, *Ehrlichia* and *Anaplasma*, neglecting other groups, especially protozoa [44]. For example, *Rh. linnaei* is a tick closely associated with domestic canids and is also recognized as a highly anthropophagous ectoparasite [45]. Since this tick has an intra-domiciliary life cycle and is in close contact with human populations, it poses a potential risk for the pathogen’s transmission. The confirmation of *Rh. sanguineus* s.s. in Guanajuato is a significant finding, as it indicates the range of expansion of this tick species in Mexico [46]. This tick was previously documented in the northern States of Baja California, Sonora, and Chihuahua, and has now been detected in central Mexico, which highlights its adaptability to a wide range of habitats [33,47]. This geographic expansion raises concerns about the potential for the increased transmission of tick-borne diseases, as *Rh. sanguineus* s.s. is a known vector for pathogens such as *Rickettsia massiliae* and *Ehrlichia canis* (causing *Rickettsia massiliae*-spotted fever and canine monocytic ehrlichiosis) [33]. The sympatry of *Rh. sanguineus* with *Rh. linnaei* in Guanajuato is particularly noteworthy, as the two species have distinct vectorial competencies. Their coexistence in the same environment needs further research to understand the implications for pathogen transmission dynamics, especially in areas where both ticks may be present on the same hosts. This study underscores the importance of ongoing surveillance and research into the ecological and epidemiological impacts of tick species distribution in Mexico [48].

Further, *Trypanosoma caninum* is one of the most recently recognized species of the genus, which was first described in Brazil in 2009 from dog skin samples [49,50,51,52,53,54]. Although it is apparently restricted to domestic canids in Brazil, a related species was found in *Ixodes ricinus* from Slovakia in recent years [55]. Our study provides the first record of this species of trypanosomatid DNA in *Rh. linnaei* ticks worldwide. However, it is still premature to establish a role for this tick in the life cycle of the parasite, since the individuals examined were collected from dogs, so it cannot be established whether the parasite DNA was naturally present in the tick or whether it came from the feeding of an infected dog. Therefore, it is a priority to conduct studies to evaluate the vector competence of this hard tick species. Although *T. caninum* is considered a non-pathogenic species, it is important to understand its life cycle and the possible immune response it exerts on the host. In an experimental study conducted in Brazil, it was shown that sera from dogs diagnosed with *T. cruzi* infection did not show cross-reactivity with *T. caninum* lysates [56]. However, this phenomenon should be evaluated in larger canine populations, as well as in humans, particularly in communities with a high abundance and biting rate of species of the *Rh. sanguineus* s.l. complex, to identify the degree of cross-reactivity and the possible confounding effect that may occur in sero-epidemiological surveys [51,57].

The telmophagous habits of this tick, which degrade the outer layer of the skin by using various enzymes, facilitate the exposure to *T. caninum* that lives in the dermis of the dog [49,58]. Previous studies with other species such as *Trypanosoma rhipicephalis* have demonstrated the trans-ovarian transmission of these protozoa in tick progeny [59]. Future studies should monitor the presence of genetic material and characteristic forms of trypanosomatids in *Rh. linnaei* populations.

Although *T. cruzi* DNA has been detected in brown dog ticks (*Rh. sanguineus* s.s.) associated with dogs from Chile [27], the role of ticks as potential vectors has not been explored to date.

Further, *Parabodo caudatus* was first isolated from a human urine sample in Czechoslovakia in 1950 [60]. However, its role as a symbiont or pathogen has not been widely discussed. It was suspected that it was an agent that contaminated urine samples from humans, although a recent report detected the presence of this protozoan in the urine of a laboratory dog with haematuria [60]. This possibility was supported in a recent trial in which its presence was detected in the blood of domestic canines living in Bangkok [61]. A similarity of 96% is too low to define a kinetoplastid species, so we decided to name the detected lineage as *Parabodo* sp. Additionally, the phylogenetic analysis showed that there are several branches that could indicate that they may be different species, not yet formally described. Since the knowledge of the *Parabodo* genus is very limited, several sequences that have been deposited as *P. caudatus* in GenBank may represent different (not yet described) species, mainly because this is a database where there is no curation of information. Therefore, it is possible to consider that, in a more solid taxonomic study, it would be concluded that these *P. caudatus* sequences are, in fact, sequences from different species close to this species. Additionally, since *P. caudatus* was detected from urine samples in a dog, it is possible to suspect that the microorganism detected in the present study, as well as in dog blood in Bangkok [61], corresponds to an undescribed species, which requires priority in further studies. Since our study now reports *Parabodo* DNA in ticks, it is tempting to propose that the parasite possibly remains in dogs’ blood circulation long enough for an ectoparasite, such as *Rh. linnaei*, to acquire it.

The species belonging to the subfamily Blechomonadinae have only been described from the Palearctic and Afrotropical regions [10]. *Blechomonas lauriereadi* has been isolated and described from the hindgut of the fleas *Ct. canis* and *Ct. felis felis* from *Vulpes vulpes* in the Czech Republic in 2013 [10]. Our study represents the first record of *B. lauriereadi* outside the Palearctic region and the first for the American continent, which notoriously increases the geographic distribution of this trypanosomatid species. Additionally, it expands the range of vertebrate host species that harbor infected fleas by documenting positive results for *Ct. felis felis* collected in dogs, given that this species of fleas has a cosmopolitan distribution [62]. Clearly, it is a priority to implement studies on these ectoparasites to determine the distribution and prevalence of this protozoan in *Ct. felis felis* populations worldwide. Additionally, it is a priority to evaluate whether this species can infect dogs, since there are no previous studies aimed at monitoring blood and/or skin samples from canids that are hosts of fleas testing positive for protozoan pathogens.

The findings of this study increase the inventory of trypanosomatids associated with ectoparasites of dogs in Mexico and the American continent, as well as the inventory of this family nationwide. Further, the presence of two genera, *Parabodo* and *Blechomonas*, are documented for the first time in Mexico.

## 5. Conclusions

The present study demonstrates the detection of DNA from three kinetoplastid species, which may be the result of the presence of these parasites in these ectoparasites and/or in the blood of the animals on which they fed. In this sense, two approaches should be taken in future studies: (1) separate the digestive tract of fleas and ticks and perform the diagnosis separately. Detection of DNA outside the digestive tract may be an indication of infection; (2) experimental studies with artificial infected blood feeding with the subsequent follow-up of their detection in the ectoparasites, both by dissection and direct observation, and molecular diagnosis. Due to the challenges of dissecting ectoparasites while preventing cross-contamination of their organs with pathogen DNA, alternative methods to PCR should be considered. Immunohistochemistry, for instance, can be employed to precisely identify the pathogen′s localization within specific anatomical sites of the ectoparasite. Similar experimental strategies have been described for *Mycoplasma* surveillance in ticks [63].

Given the limited knowledge of the detected kinetoplastid species, it is a priority to establish studies aimed at identifying the biology, ecology, biogeography, and epidemiology of these agents to understand their life history and contextualize them as part of the biodiversity of protozoa nationwide.

## Figures and Tables

**Table 1:** Collection sites of ticks and fleas associated with domestic animals and people in Mexico. Abbreviations: BL: *Blechomonas lauriereadi*; ND: Not determined; TC: *Trypanosoma caninum*; PS: *Parabodo* sp.

State	Municipality	Locality	Coordinates	Host	Collection Date	Ectoparasite Species	Total	Female	Male	Nymph	TC	PS	BL
Aguascalientes	Aguascalientes	Peñuelas	21°43′44′′ N, 102°16′19′′ W	3 Dogs	28 July 2021	*Ctenocephalides felis felis*	4	2	2	0	−	−	−
				2 Dogs	28–29 July 2021	*Pulex simulans*	5	4	1	0	−	−	−
	Calvillo	Calvillo	21°50′49″ N, 102°42′52″ W	2 Dogs	15,20 May 2021	*Rhipicephalus linnaei*	8	5	3	0	−	−	−
		Ventanillas	21°57′53″ N, 102°40′59″ W	4 Dogs	20 August 2021	*Rh. linnaei*	4	2	2	0	−	−	−
	Rincón de Romos	Rincón de Romos	22°13′44″ N, 102°18′18″ W	6 Dogs	28 April 2021	*Rh. linnaei*	10	1	7	2	−	−	−
Baja California	Mexicali	Colonia Hidalgo	32°36′54″ N, 115°27′03″ W	1 Dog	09 September 2019	*Rh. linnaei*	2	0	2	0	−	−	−
		Ejido Cuernavaca	32°33′52″ N, 115°18′11″ W	9 Dogs	09 September 2019	*Rh. linnaei*	16	6	10	0	−	−	−
		Ejido Sinaloa	32°32′51″ N, 115°16′11″ W	4 Dogs	19 September 2019	*Rh. linnaei*	6	4	2	0	−	−	−
Campeche	Champotòn	Champotòn	19°20′40″ N, 90°43′28″ W	19 Dogs	12 August 2021	*Rh. linnaei*	26	19	7	0	−	−	−
Chihuahua	Chihuahua	Colonia El Porvenir	31°14′22″ N, 105°52′31″ W	2 Human	04 February 2020	*Rh. linnaei*	2	0	2	0	+	−	−
		Colonia Tarahumara	28°44′05″ N, 106°06′45″ W	6 Dogs	28 August 2019	*Rh. linnaei*	6	6	0	0	−	−	−
		Colonia Zaarco	28°36′43″ N, 106°04′55″ W	1 Human	25 September 2023	*Rh. linnaei*	1	1	0	0	−	−	−
		Chihuahua	28°38′13″ N, 106°04′37″ W	1 Human	27 January 2020	*Rh. linnaei*	1	1	0	0	−	−	−
		Granjas del Valle	28°45′10″ N, 106°7′3″ W	3 Human	19/09/2023	*Rh. linnaei*	6	5	1	0	−	−	−
		Rinconada los Nogales	28°37′27″ N, 105°59′40″ W	1 Human	11 February 2020	*Rh. linnaei*	1	0	1	0	−	−	−
	Juárez	ND	28°36′39″ N, 106°00′51″ W	4 Dogs	14–15 August 2019	*Rh. linnaei*	4	4	0	0	−	−	−
	Meoqui	Lázaro Cárdenas	28°23′23″ N, 105°37′25″ W	1 Human	11 August 2023	*Rh. linnaei*	2	2	0	0	−	−	−
Guanajuato	Comonfort	Comonfort	20°42′54″ N, 100°45′24″ W	9 Dogs	23–24 May 2019	*Rhipicephalus sanguineus*	16	4	7	5	−	−	−
	Purísima	Purísima	21°01′50″ N, 101°52′45″ W	9 Dogs	23 May 2023	*Rh. linnaei*	15	7	8	0	+	−	−
	San Miguel de Allende	San Miguel de Allende	20°54′55″ N, 100°44′38″ W	2 Dogs	31 May 2019	*Rh. sanguineus*	3	1	2	0	−	−	−
Hidalgo	Ixmiquilpan	La Reforma	20°28′42″ N, 99°13′52″ W	2 Dogs	05 April 2019	*Rh. linnaei*	2	2	0	0	−	−	−
	Huazalingo	Tzapotitla	20°58′46″ N, 98°28′55″ W	2 Dogs	15 August 2019	*Amblyomma mixtum*	2	0	2	0	−	−	−
Jalisco	Puerto Vallarata	Mandarina	20°31′51″ N, 105°17′32″ W	ND	ND	*A. mixtum*	11	4	3	4	−	−	−
						*Rh. linnaei*	2	2	0	0	−	+	−
Mexico City	Iztacalco	Campamento 2 de Octubre	19°23′05″ N, 99°06′59″ W	1 Human	06 January 2023	*A. mixtum*	1	1	0	0	−	−	−
Morelos	Emiliano Zapata	El Calvario	18°50′58″ N, 99°10′57″ W	1 Dog	05 February 2019	*Ct. felis felis*	3	3	0	0	−	−	−
	Puente de Ixtla	Xoxocotla	18°41′06″ N, 99°14′38″ W	5 Dogs	05 March 2019	*Rh. linnaei*	11	2	4	5	−	+	−
	Temixco	Lomas del Carril	18°51′55″ N, 99°14′00″ W	4 Dogs	19 February 2019; 14 February 2024	*Ct. felis felis*	18	12	6	0	−	−	−
				12 Dogs	17 December 2018; 13 February 2019; 10 March 2019	*Rh. linnaei*	17	8	9	0	−	+	−
	Xochitepec	Loma Bonita	18°56′02″ N, 99°10′23″ W	1 Dog	30 January 2019	*Ct. felis felis*	3	2	1	0	−	−	−
Nayarit	Compostela	Chacala	21°10′00″ N, 105°13′28″ W	ND	ND	*Rh. linnaei*	15	6	6	3	+	−	−
Nuevo León	Allende	Comunidad los Sabinos	25°19′06.903″ N, 99°59′09.816″ W	1 Dog	02 September 2021	*Ct. felis felis*	5	3	2	0	−	−	+
	Cadereyta	Valle del Roble	25°36′08″ N, 100°04′32″ W	3 Dogs	10 September 2021	*Rh. linnaei*	6	4	2	0	−	−	−
	Dr. Arroyo	La Chiripa	25°37′56″ N, 100°10′14″ W	2 Dogs	21 September 2021	*Rh. linnaei*	4	3	1	0	−	−	−
		Jesús María de Berrones	23°55′02″ N, 100°01′49″ W	3 Dogs	02 September 2021	*Rh. linnaei*	4	3	1	0	−	−	−
		La Unión y El Cardonal	23°50′51″ N, 100°05′29″ W	2 Dogs	14 September 2021	*Rh. linnaei*	3	1	2	0	−	−	−
	Escobedo	Colinas del Topo	25°47′50″ N, 100°22′27″ W	2 Dogs	13 August 2021	*Rh. linnaei*	5	4	1	0	−	−	−
		Fuentes de Escobedo	25°47′14″ N, 100°18′41″ W	Environment	31 August 2021	*Rh. linnaei*	8	1	7	0	−	−	−
		Palmiras	25°50′36″ N, 100°25′22″ W	2 Dogs	09 August 2021	*Rh. linnaei*	3	1	2	0	−	−	−
		Pedregal de Topo Chico	25°47′05″ N, 100°19′42″ W	2 Dogs	14 September 2021	*Rh. linnaei*	4	2	2	0	−	−	−
		Santa Lucía	25°48′10″ N, 100°22′01″ W	2 Dogs	13 August 2023	*Rh. linnaei*	4	2	2	0	−	−	−
		Villas de Francisco	25°47′41″ N, 100°20′12″ W	2 Dogs	20 September 2021	*Rh. linnaei*	4	2	2	0	−	−	−
	García	Paseo de las Torres	25°47′20″ N, 100°35′50″ W	Environment	14 September 2021	*Rh. linnaei*	2	2	0	0	−	−	−
	General Zuazua	Valle de Santa Elena	25°51′18″ N, 100°08′32″ W	4 Dogs	08 September 2021	*Rh. linnaei*	6	2	4	0	−	−	−
	Guadalupe	Jardínes de Santa Clara	25°40′09″ N, 100°12′34″ W	1 Dog	12 August 2023	*Rh. linnaei*	1	0	1	0	−	−	−
		Residencial Dos Ríos	25°40′39″ N, 100°15′35″ W	2 Dogs	25 August 2021	*Rh. linnaei*	2	2	0	0	−	−	−
		Tierra Propia	25°38′23″ N, 100°11′01″ W	5 Dogs	26 August 2021	*Rh. linnaei*	5	3	2	0	−	−	−
		Unidad Reforma	25°40′08″ N, 100°08′00″ W	2 Dogs	27 August 2021	*Rh. linnaei*	2	0	2	0	−	−	−
	Juárez	Real de San Josè	25°39′28″ N, 100°04′56″ W	8 Dogs	29 March 2021	*Rh. linnaei*	8	6	2	0	−	−	−
		Terranova	25°39′41″ N, 100°06′07″ W	3 Dogs	15 July 2021	*Rh. linnaei*	3	2	1	0	−	−	−
	Linares	Ejido San José	25°47′41″ N, 100°27′49″ W	1 Dog	14 April 2021	*Amblyomma tenellum*	10	9	1	0	−	−	−
		Villegas	25°51′27″ N, 99°33′38″ W	11 Dogs	12 August 2021	*Rh. linnaei*	15	8	7	0	−	−	−
	Mina	El Pedregal	26°00′13″ N, 100°31′57″ W	2 Dogs	21 September 2021	*Rh. linnaei*	3	1	2	0	−	−	−
	Montemorelos	Maranatha	25°11′50″ N, 99°51′16″ W	1 Dog	15 April 2021	*A. tenellum*	1	1	0	0	−	−	−
	Monterrey	Centro	25°40′31″ N, 100°18′42″ W	1 Dog	07 September 2021	*Rh. linnaei*	4	1	3	0	−	−	−
		Los Nogales	25°44′02″ N, 100°19′19″ W	11 Dogs	08 July 2021; 14 August 2021; 24 August 2021	*Rh. linnaei*	18	9	9	0	−	−	−
		Morelos	25°43′14″ N, 100°20′53″ W	1 Dog	27 July 2021	*Rh. linnaei*	6	3	3	0	−	−	−
		San Antonio	25°39′26″ N, 100°17′30″ W	4 Dogs	22 July 2021	*Rh. linnaei*	6	6	0	0	−	−	−
		Terminal	25°41′16″ N, 100°17′50″ W	2 Dogs	11 August 2021	*Rh. linnaei*	4	2	2	0	−	−	−
		Valle de Santa Lucía	25°44′41″ N, 100°21′20″ W	3 Dogs	24 August 2021	*Rh. linnaei*	3	3	0	0	−	−	−
	San Nicolás de los Garza	Constituyentes de Querétaro	25°43′02″ N, 100°15′09″ W	2 Dogs	13 September 2021	*Rh. linnaei*	4	2	2	0	−	−	−
		San Cristobal	25°44′07″ N, 100°12′49″ W	2 Dogs	15 September 2021	*Rh. linnaei*	3	1	2	0	−	−	−
	Santa Catarina	Enrique Rangel	25°44′39″ N, 100°21′09″ W	2 Dogs	09 September 2021	*Rh. linnaei*	4	2	2	0	−	−	−
		San Gilberto	25°41′53″ N, 100°27′37″ W	2 Dogs	15 September 2002	*Rh. linnaei*	2	1	1	0	−	−	−
Puebla	Atlixco	Tolometla	18°55′34″ N, 98°23′52″ W	1 Dog	10 May 2019	*Ct. felis felis*	4	4	0	0	−	−	+
	San Pedro Yeloixtlahuaca	San Juan Llano Grande	18°03′50″ N, 98°05′04″ W	5 Dogs	20 November 2019	*Rh. linnaei*	10	6	4	0	−	−	−
											−	−	−
	Santiago Miahuatlán	San Isidro Labrador	18°32′30″ N, 97°26′27″ W	11 Dogs	28 June 2019–13/September/2019	*Rh. linnaei*	18	10	8	0	−	−	−
	Tehuacán	Tehuacán	18°27′29″ N, 97°24′26″ W	14 Dogs, 1 Cat	11, 14, 20, 22, 24 November 2019; 08 December 2023	*Rh. linnaei*	31	13	18	0	−	−	−
				5 Dogs	24 November 2019	*Ct. felis felis*	14	8	6	0	−	−	+
	Tenampulco	Tenampulco	20°10′11″ N, 97°24′23″ W	6 Dogs	03 March 2021	*Rh. linnaei*	10	5	5	0	−	−	−
	Tepexi de Rodríguez	Estrella Roja	18°34′45″ N, 97°55′39″ W	3 Dogs	21 May 2019	*Rh. linnaei*	4	2	2	0	−	−	−
	Tlacotepec de Benito Juárez	Tlacotepec de Benito Juárez	18°40′35″ N, 97°39′03″ W	3 Dogs	04 December 2019	*Ct. felis felis*	12	8	4	0	−	−	−
	Venustiano Carranza	Estrella Roja	20°30′18″ N, 97°40′15″ W	1 Dog	21 May 2019	*Rh. linnaei*	1	1	0	0	−	−	−
	Zongozotla	Zotik	19°58′52″ N, 97°43′44″ W	2 Dogs	11 June 2019	*Ct. felis felis*	3	0	3	0	−	−	−
San Luis Potosí	Lagunillas	Mirador	21°53′40″ N, 100°51′13″ W	1 Dog	07 September 2023	*Rh. linnaei*	2	0	2	0	−	−	−
Sinaloa	Ahome	Nuevo San Miguel	25°57′52″ N, 109°03′22″ W	7 Dogs	17 March 2021	*Rh. linnaei*	20	7	13	0	−	−	−
Sonora	Agua Prieta	Agua Prieta	31°17′44″ N, 109°32′29″ W	2 Dogs	27 June 2019	*Rh. linnaei*	8	2	6	0	+	−	−
	Hermosillo	Hermosillo	29°05′00″ N, 110°59′17″ W	5 Dogs	07 September 2023	*Rh. Linnaei*	7	4	3	0	+	−	−
	Imuris	Imuris	30°47′21″ N, 110°50′37″ W	4 Dogs	03 July 2019	*Rh. Linnaei*	8	2	6	0	−	−	−

## Data Availability

The data that support the results of this study are available in GenBank, under the following accession numbers Kinetoplastids (PQ496669-PQ496680) and *Rhipicephalus sanguineus* s.l. (PQ495570-PQ495590).
